# The Ability of Respiratory Commensal Bacteria to Beneficially Modulate the Lung Innate Immune Response Is a Strain Dependent Characteristic

**DOI:** 10.3390/microorganisms8050727

**Published:** 2020-05-13

**Authors:** Ramiro Ortiz Moyano, Fernanda Raya Tonetti, Mikado Tomokiyo, Paulraj Kanmani, María Guadalupe Vizoso-Pinto, Hojun Kim, Sandra Quilodrán-Vega, Vyacheslav Melnikov, Susana Alvarez, Hideki Takahashi, Shoichiro Kurata, Haruki Kitazawa, Julio Villena

**Affiliations:** 1 Laboratory of Immunobiotechnology, Reference Centre for Lactobacilli (CERELA-CONICET), 4000 Tucumán, Argentina; rortiz@cerela.org.ar (R.O.M.); salvarez@cerela.org.ar (S.A.); 2 Infection Biology Laboratory, Instituto Superior de Investigaciones Biológicas (INSIBIO), CONICET-UNT, 4000 Tucumán, Argentina; frayatonetti@gmail.com (F.R.T.); mgvizoso@fm.unt.edu.ar (M.G.V.-P.); 3 Laboratorio de Ciencias Básicas & Or. Genética, Facultad de Medicina, Universidad Nacional de Tucumán, 4000 Tucumán, Argentina; 4 Food and Feed Immunology Group, Laboratory of Animal Products Chemistry, Graduate School of Agricultural Science, Tohoku University, Sendai 981-8555, Japan; mikado0403@gmail.com; 5 Livestock Immunology Unit, International Education and Research Center for Food and Agricultural Immunology (CFAI), Graduate School of Agricultural Science, Tohoku University, Sendai 981-8555, Japan; 6 Department of Korean Medicine, Dongguk University, Goyang 410-050, Korea; kanmanibiotech2007@gmail.com (P.K.); kimklar@dongguk.ac.kr (H.K.); 7 Laboratory of Food Microbiology, Faculty of Veterinary Sciences, University of Concepción, 3780000 Chillán, Chile; squilodran@udec.cl; 8 Gabrichevsky Research Institute for Epidemiology and Microbiology, 125212 Moscow, Russia; goutch@mail.ru; 9 Laboratory of Plant Pathology, Graduate School of Agricultural Science, Tohoku University, Sendai 981-8555, Japan; hideki.takahashi.d5@tohoku.ac.jp; 10 Plant Immunology Unit, International Education and Research Center for Food and Agricultural Immunology (CFAI), Graduate School of Agricultural Science, Tohoku University, Sendai 981-8555, Japan; 11 Laboratory of Molecular Genetics, Graduate School of Pharmaceutical Sciences, Tohoku University, Sendai 981-8555, Japan; kurata@mail.pharm.tohoku.ac.jp

**Keywords:** respiratory commensal bacteria, *Dolosigranulum pigrum*, *Corynebacterium pseudodiphtheriticum*, TLR2, TLR3, respiratory syncytial virus, *Streptococcus pneumoniae*

## Abstract

We investigated whether the ability of commensal respiratory bacteria to modulate the innate immune response against bacterial and viral pathogens was a shared or strain-specific characteristic. Bacterial strains belonging to the *Corynebacterium pseudodiphtheriticum* and *Dolosigranulum pigrum* species were compared by studying their influence in the Toll-like receptor (TLR)-2- and TLR3-triggered immune responses in the respiratory tract, as well as in the resistance to Respiratory Syncytial Virus (RSV) and *Streptococcus pneumoniae* infections. We demonstrated that nasally administered *C. pseudodiphteriticum* 090104 or *D. pigrum* 040417 were able to modulate respiratory immunity and increase the resistance against pathogens, while other strains of the same species did not influence the respiratory immune responses, demonstrating a clear strain-dependent immunomodulatory effect of respiratory commensal bacteria. We also reported here that bacterium-like particles (BLP) and cell walls derived from immunomodulatory respiratory commensal bacteria are an interesting alternative for the modulation of the respiratory immune system. Our study is a step forward in the positioning of certain strains of respiratory commensal bacteria as next-generation probiotics for the respiratory tract.

## 1. Introduction

Respiratory infections are a common cause of morbidity and mortality. More than 4 million people die yearly as a consequence of acute respiratory infections worldwide [1]. Globally, human respiratory syncytial virus (RSV) and *Streptococcus pneumoniae* are the most important cause of fatal respiratory infections, especially in high-risk populations such as infants and children. Generally, RSV infections are self-limiting and restricted to the upper airways. However, in susceptible individuals, the virus may spread to the lower tract causing more severe symptoms. The viral respiratory attack may turn immune response into pathological resulting in the loss of function and even death. In addition, clinical and epidemiologic data suggest that RSV is linked to increases in the frequency [2] and severity [3] of pneumococcal disease. It was demonstrated in animal models that RSV infection before pneumococcal challenge or the simultaneous infection with both respiratory pathogens significantly increases lung injury and the incidence of bacteremia [4,5]. RSV infection produces a local destruction of the epithelium, induces respiratory ciliary dyskinesia [5], up-regulate the expression of adhesion factors in respiratory epithelial and endothelial cells [6], and impairs the innate defenses [4,5,7] favoring pneumococci for colonization and spread. In addition, it was reported that the direct interaction between RSV and *S. pneumoniae* induce modifications in the transcriptome of the bacterial pathogen leading to an enhanced expression of the virulence factors neuraminidase A/B and pneumolysin, potentiating the infectivity of pneumococci [5]. Those findings highlight the complex interactions that exist between RSV and *S. pneumoniae* and the host, which must be efficiently regulated in order to diminish the severity and mortality of respiratory infections caused by these pathogens. In this regard, taking into consideration the increased antibiotic resistance of pneumococci and that the therapeutic possibilities for the treatment of viral infections are directed to reducing the symptoms but are not effective to fight off the virus; novel approaches to prevent respiratory infections and superinfections are urgently needed.

The recent advances in “omics” sciences have allowed the discovery of niche-specific communities of microorganisms in the human gastrointestinal tract that have been associated with health promoting effects. Moreover, the isolation and functional characterization of these beneficial gastrointestinal commensal microorganisms has opened the door to a new kind of probiotics termed “next-generation probiotics”, which have started to be used to restore a healthy homeostasis within the gastrointestinal tract [8]. In this regard, new next-generation probiotic bacteria such as *Bacteroides fragilis* [9], *Faecalibacterium prausnitzii* [10] and *Akkermansia municiphila* [11], have been associated with a beneficial modulation of the gastrointestinal immune response. More recently, niche-specific communities of microorganisms have been also described in the respiratory tract that spans from the nostrils to the lung alveoli [12]. Moreover, it was suggested that a healthy respiratory microbiota may influence the pathogenesis of respiratory diseases affecting for example the outcome of respiratory tract bacterial infections [13]. Although the respiratory microbiota has not been studied as deeply as the intestinal microbiota, several reports evidence that certain bacteria positively influence the respiratory health of the host making possible the study and characterization of new “next-generation probiotics” for the respiratory tract. 

Corynebacterium and Dolosigranulum belong to the main beneficial members of the nasopharynx microbiota [14], and several lines of evidence suggest that both species of bacteria play a protective role in the upper respiratory tract. Mean levels of Corynebacterium and Dolosigranulum were correlated and were protective against *S. pneumoniae* colonization [15,16]. Moreover, it was demonstrated that *Dolosigranulum* spp. reduced the risk of acquiring respiratory infections such as invasive disease caused by *S. pneumoniae* [17]. In addition, the beneficial effects of respiratory commensal bacteria against viral respiratory infections have been reported recently. *Dolosigranulum* spp. reduced the risk of flu by Influenza A [18], while our previous study showed that *Corynebacterium pseudodiphtheriticum* strain 090104 had protective properties improving mice resistance to RSV and *S. pneumoniae* superinfection through the modulation of the respiratory innate immune response [19]. 

The effect of probiotics on the immune system have been shown to be a strain-specific property since the immunomodulatory activity of one strain cannot be extrapolated to others even of the same species [20]. Therefore, this fact and the previous findings described above motivated us to study whether the ability of commensal respiratory bacteria to modulate the innate immune response against bacterial and viral pathogens was a shared or strain-specific characteristic. We also evaluated whether the viability of commensal respiratory bacteria was necessary to obtain the optimal beneficial effect on the respiratory innate immune response and the protection against pathogens.

## 2. Materials and Methods 

### 2.1. Microorganisms 

*Dolosigranulum pigrum* (*D. pigrum*) and *Corynebacterium pseudodiphteriticum* (*C. pseudodiphteriticum*) strains were cultured 18 h at 37 °C (late log phase) in trypticase soy broth. Bacteria suspensions were prepared as described previously [19]. Briefly, cultures were harvested by centrifugation at 3000× *g* for 10 min, washed three times with sterile 0.01 M phosphate buffer saline (PBS, pH 7.2) and resuspended in sterile PBS. Differences between the strains within a same species was evaluated by sequencing and comparison of 16s RNA (data not shown).

Bacterium-like particles (BLPs) from *C. pseudodiphteriticum* 090104 and *D. pigrum* 040417 were obtained by following the method of [21]. Briefly, bacteria from a fresh overnight culture (100 mL) were collected by centrifugation (10 min, 13,000× *g*) and washed once with sterile distilled water. Afterwards, the pellet was suspended in 20 mL of 0.1 M HCl and boiled in a water bath for 45 min. Next, the cells were washed three times in 50 mL sterile PBS, pH 7.4, with vigorous vortexing. After washing, the cells were resuspended in 10 mL PBS and stored at −20 °C. The number of BLPs per milliliter was adjusted according to the CFU/mL determined in the starting culture. The lack of viable bacteria within the BLPs preparation was checked by plating the suspensions and several dilutions on to BHI agar plates, which were incubated 18 h at 37 °C.

Cell wall from *D. pigrum* 040417 was obtained using the method of Shida et al. [22] with minor modifications. Briefly, the grown bacterium was washed three times with sterile PBS and lyophilized. Then, the cells were resuspended in sterile water (0.1 g/mL) and were lysed by sonication in an Ultrasonic Homogenizer (Branson Ultrasonics, Fisher Scientific, Waltham, MA, USA) with cycles of 2.5 min and an amplitude of 70%. The wall obtained in this way was delipidated by successive refluxing with methanol, methanol-chloroform (1:1), and chloroform. The delipidated preparation was resuspended in Tris-HCl buffer pH 50 µM 7.2 to 7.5 and treated with bovine pancreatic DNAse I (Sigma-Aldrich) (50 µg/mL) and ribonuclease A (Sigma-Aldrich, San Luis, MO, USA) (100 µg/mL) at 37 °C with stirring for 4 h. The insoluble material was washed with distilled water and lyophilized; the resultant product was used as the cell wall preparation. 

### 2.2. Animals and TLRs Agonist Administration 

Three-week-old BALB/c female mice were obtained from the closed colony kept at CERELA (San Miguel de Tucumán, Argentina). This study was carried out in strict accordance with the recommendations in the Guide for the Care and Use of Laboratory Animals of the Guidelines for Animal Experimentation of CERELA. The CERELA Institutional Animal Care and Use Committee prospectively approved this research under the protocol BIOT-CRL-18 (approved on 5 May 2018). All efforts were made to minimize the number of animals and their suffering. No signs of discomfort or pain were observed before mice reached the endpoints. No deaths were observed before mice reached the endpoints.

During the experiments, mice were individually housed in plastic cages at room temperature. Viable *C. pseudodiphteriticum* or *D. pigrum* strains as well as BLPs from *C. pseudodiphteriticum* 090104 and *D. pigrum* 040417 were nasally administered to different groups of mice for 5 consecutive days at a dose of 10^8^ bacterial particles/mouse/day in 50 µL of PBS. An additional group of mice was nasally treated with the cell wall of *D. pigrum* 040417 for 5 consecutive days at a dose of 8 µg/mL per day in 50 µL of PBS (CWDP group). The priming protocols were selected by assessing several doses and administration times (data not shown). One day after the last administration of viable bacteria, BLPs or CWDP, mice were nasally challenged with poly(I:C) as described previously [19,23]. Briefly, mice were lightly anesthetized and 50 µL of PBS, containing 250 µg poly(I:C) (equivalent to 10 mg/kg body weight), was administered. Mice received three doses of poly(I:C) (LMW, Sigma-Aldrich, San Luis, MO, USA) with 24 h rest period between each administration. In a second set of experiments, one day after the last administration of viable bacteria, BLPs or CWDP, mice were nasally challenged with 50 µL of a mixture of the synthetic bacterial lipopeptide analog Pam3CSK4 (InvivoGen, San Diego, CA, USA, 600 µg/mL) and the macrophage-activating lipopeptide 2 KDa (MALP2, 300 µg/mL). Mice received two doses of MALP2/Pam3CSK4 with 24 h rest period between each administration. In both sets of experiments, the treated groups and the untreated control mice were fed a conventional balanced diet ad libitum.

### 2.3. Respiratory Syncytial Virus and Streptococcus Pneumoniae Infections 

Vero cells were infected with Human RSV strain A2 at a multiplicity of infection (MOI) of 1 in 5 mL of Dulbecco’s modified Eagle’s medium (DMEM). Cells were infected for 2.5 h at 37 °C and 5% CO_2_. After infection, 7 mL of DMEM with 10% FBS (Sigma, Tokyo, Japan), 0.001% ciprofloxacin (Bayer) and 0.1% penicillin–streptomycin (Pen/Strep) (Sigma, Tokyo, Japan) were added to the flask and cells were incubated until extensive syncytium formation was detected. Then, Vero cells were scraped, lysated and the cell lysates were centrifuged at 700× *g* for 10 min at 4 °C to remove cell debris. Cell-free virus stocks (5 × 10^8^ PFU RSV) were stored in 30% sucrose at −80 °C.

For in vivo RSV infection, mice were slightly anesthetized and intranasally challenged with 2.4 × 10^6^ PFU RSV strain A2 in 50 µL. RSV immune plaque assay was performed to evaluate the viral infection as described previously [19,23]. Briefly, intact lung tissue was removed and stored in 30% sucrose. Afterwards, lungs were homogenized using a pellet pestle and centrifuged at 2600× *g* for 10 min at 4 °C to clarify the supernatant. Vero cells (1.5 × 10^5^ cells/well) were plated in in DMEM medium containing 10% FBS, 0.1% Pen/Strep, and 0.001% ciprofloxacin. All samples were seeded onto 24-well tissue culture plates and incubated at 37 °C and 5% CO_2_ for 2.5 h. Then, the supernatant was removed after incubation, and 1 mL of fresh DMEM medium (10% FBS, 0.1% Pen/Strep, and 0.001% ciprofloxacin) was added to the monolayers. When extensive syncytia developed, the overlay was removed and wells were fixed with ice-cold acetone:methanol (60:40). Then, the monolayers were treated with primary anti-RSV-F (clones131-2A; Chemicon International Inc., Temecula, CA, USA) and anti-RSV-G (Mouse monoclonal [8C5 (9B6)] to RSV glycoproteins, Abcam, Cambridge, UK) antibodies for 2 h, followed by secondary horseradish peroxidase anti-mouse immunoglobulin antibody (Anti-mouse IgG, HRP-linked Antibody #7076, Cell signaling Technology; Danvers, MA, USA) for 1 h. Plates were washed twice with PBS containing 0.5% Tween 20 (Sigma-Aldrich, San Luis, MO, USA) after each antibody incubation step. Individual plaques were developed using a DAB substrate kit (ab64238, Abcam, Cambridge, UK) following the manufacturer’s instructions. Results were expressed as log_10_ PFU/g of lung.

*S. pneumoniae* serotype 6B was first grown on blood agar for 18 h. Freshly grown colonies were suspended in Todd Hewitt broth (Oxoid, Cambridge, UK) and incubated overnight at 37 °C. The pathogen was harvested by centrifugation at 3600× *g* for 10 min, and then washed three times with sterile PBS. Cell density was adjusted to 1 × 10^9^ CFU/mL. The size of the inoculum in challenge experiments was confirmed by serial dilutions and quantitative subcultures on blood agar as described previously [24].

Mice were challenged nasally with 10^6^ CFU of the pathogen per mouse. Animals were infected by dropping 12.5 µL of *S. pneumoniae* in PBS into each nostril, which was then involuntarily inhaled. To facilitate migration of the inoculum to the alveoli, mice were held in a head-up vertical position for 2 min. Mice were sacrificed 2 days after the challenge. Lungs were aseptically excised, weighed, and homogenized in 5 mL of sterile peptone water. Homogenates were diluted appropriately, plated in duplicate on blood agar, and incubated for 18 h at 37 °C. *S. pneumoniae* colonies were counted and results were expressed as log_10_ CFU/g of organ. The presence of bacterial growth in the bloodstream was monitored by obtaining blood samples by cardiac puncture with a heparinized syringe. Samples were plated on blood agar and bacteremia was reported as negative or positive hemocultures after incubation for 18 h at 37 °C.

### 2.4. Cytokine Concentrations in Broncho-Alveolar Lavages (BAL) 

BAL samples were obtained as described previously [19,23]. Briefly, the trachea was exposed and intubated with a catheter and two lavages of lungs with sterile PBS were performed. After centrifugation, cell-free supernatants were kept at −70 °C until analysis Tumor necrosis factor (TNF)-α, interferon (IFN)-γ, IFN-β, IFN-α, interleukin (IL)-6, and IL-10 concentrations in serum and BAL were measured by enzyme-linked immunosorbent assay (ELISA) following the manufacturer’s recommendations (R&D Systems, MN, USA).

### 2.5. Lung Cell Suspensions and Flow Cytometry Studies

Single lung cells were prepared as described previously [25]. Briefly, lungs were removed, finely chopped, and incubated for 90 min with 300 U of collagenase (Yakult Honsha Co., Tokyo, Japan) in 15 mL of RPMI 1640 medium (Sigma, Tokyo, Japan). Debris were removed and erythrocytes were depleted by hypotonic lysis. The cells were washed with RPMI medium supplemented with 0.1% Pen/Strep and suspended in a medium supplemented with 10% heat-inactivated fetal calf serum (FCS). Cells were counted using Trypan Blue and adjusted to 5 × 10^6^ cells/mL.

Lung cell suspensions were pre-incubated with anti-mouse CD32/CD16 monoclonal antibody (Fc block) for 15 min at 4 °C. Cells were incubated in the antibody mixes for 30 min at 4 °C and washed with FACS buffer. When necessary, cells were treated with Fixation/Permeabilization Solution Kit (BD Biosciences). Then, cells were stained with fluorochrome-conjugated antibodies against CD3, CD4, CD8, CD11c, CD11b, CD103, MHC-II, IFN-γ, IL-10, sialic acid binding immunoglobulin-like lectin F (SiglecF) (BD Bioscience), IFN-β, and CD45 (eBioscience, San Diego, CA, USA). Cells were then acquired on a BD FACSCaliburTM flow cytometer (BD Biosciences, Franklin Lakes, NJ, USA) and data were analyzed with FlowJo software (TreeStar). The total number of cells in each population was determined by multiplying the percentages of subsets within a series of marker negative or positive gates by the total cell number determined for each tissue [19,26].

### 2.6. Lung Tissue Injury Studies 

Albumin and protein content in cell-free BAL was quantified in order to measure increased permeability of the bronchoalveolar–capillarity barrier and lactate dehydrogenase (LDH) activity was quantified as an indicator of general cytotoxicity [23,25]. Lung wet:dry weight ratio was determined as described previously [19,26]. Briefly, mice were euthanized and exsanguinated. Their lungs were removed, weighed (wet weight), dried at 55 °C for 7 days, and weighed again (dry weight). The wet:dry weight ratio was calculated as a measure of pulmonary edema.

### 2.7. Statistical Analysis

Experiments were made in triplicate and results were expressed as mean ± standard deviation (SD). Normal distributed data were tested by 2-way ANOVA. Tukey’s test (for pairwise comparisons of the means) or the Fisher’s least significant difference (LSD) test (for multi-comparison) were used to evaluate the differences among groups. Differences were considered significant at *p* < 0.05 or *p* < 0.01. 

## 3. Results

### 3.1. Strain-Dependent Ability of Respiratory Commensal Bacteria to Modulate Lung Innate Immunity

**Figure 1:**
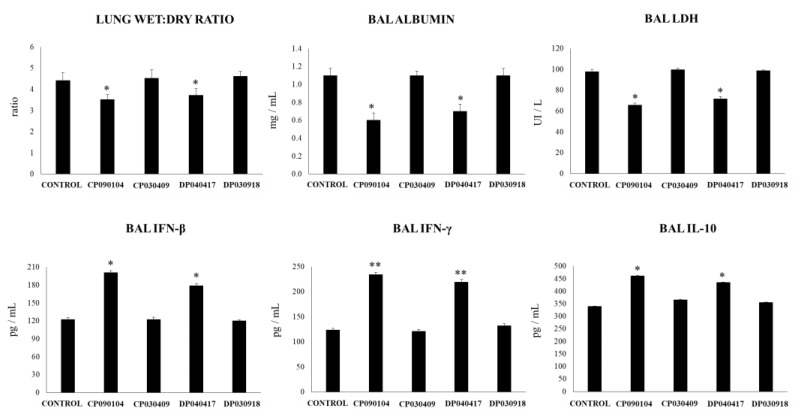
Effect of respiratory commensal bacteria strains on lung tissue damage and respiratory cytokines induced by the nasal administration of the viral pathogen-associated molecular pattern poly(I:C). Infant mice were nasally primed with viable *Corynebacterium pseudodiphteriticum* (CP) strains 090104 or 030409 or *Dolosigranulum pigrum* (DP) strains 040417 or 030918 during five consecutive days and then challenged with three once-daily doses of poly(I:C). Non-treated infant mice challenged with poly(I:C) were used as controls. Two days after the last poly(I:C) administration lung wet:dry weight ratio, lactate dehydrogenase (LDH) activity, and albumin concentrations, and the levels of interferon (IFN)-β, IFN-γ, and interleukin (IL)-10 in broncho-alveolar lavages (BAL) were determined. Experiments were performed with 5–6 mice per group. The results represent data from three independent experiments. Significantly different when compared to control * *p* < 0.05 or ** *p* < 0.01.

The nasal priming of infant mice with the respiratory commensal bacteria strains did not induce alterations in the levels of lung injury markers (Figure S1) nor modifications in the numbers of BAL macrophages or neutrophils (Figure S2A). All strains slightly increased the levels of BAL TNF-α, with *C. pseudodiphteriticum* 090104 and *D. pigrum* 040417 being the most efficient at inducing this effect (Figure S2A). In addition, only the 090104 and 040417 strains significantly increased the levels of BAL IFN-β, IFN-γ and IL-10 in basal conditions (Figure S1). We next aimed to evaluate the ability of the different respiratory commensal bacteria strains to modulate the lung inflammatory response triggered by TLR3 activation. For this purpose, infant mice were nasally primed with viable 090104, 030409 040417 or 030918 strains during five consecutive days and then challenged with three once-daily doses of poly(I:C). The inflammatory damage was studied two days after poly(I:C) challenge (Figure 1). As we describe previously [19,23], poly(I:C) administration significantly altered the lung wet:dry ratio and increased the levels of the BAL biochemical markers of injury. *C. pseudodiphteriticum* 090104 and *D. pigrum* 040417 were able to significantly reduce the changes in lung wet:dry ratio and to decrease the levels of BAL albumin and LDH, indicating their ability to reduce lung damage after TLR3 activation (Figure 1). On the contrary, mice nasally treated with *C. pseudodiphteriticum* 030409 or *D. pigrum* 030918 showed values of lung wet:dry ratio and BAL biochemical markers of injury that were no different from the control group. We previously reported that immunobiotic treatments with the ability to diminish TLR3-mediated inflammatory lung damage differentially modulate the levels of IFN-β, IFN-γ and IL-10 in the respiratory tract [19,26]. Therefore, we also evaluated the influence of the respiratory commensal bacteria strains on the concentration of these cytokines in BAL samples after poly(I:C) stimulation. TLR3 activation significantly increased the levels of BAL IFN-β, IFN-γ and IL-10 in all the experimental groups when compared to basal levels (Figure 1, Figure S1). However, mice nasally treated with *C. pseudodiphteriticum* 090104 or *D. pigrum* 040417 had values of IFN-β, IFN-γ and IL-10 in the respiratory tract that were significantly higher than those observed in the control group. The treatments with *C. pseudodiphteriticum* 030409 or *D. pigrum* 030918 were not able to modify the levels BAL IFN-β, IFN-γ or IL-10 when compared to controls. 

No differences in the numbers of BAL macrophages were found when treated mice were compared to controls. However, mice nasally treated with 090104 or 040417 strains had values of BAL TNF-α and neutrophils that were significantly higher and lower than controls, respectively (Figure S2B).

**Figure 2:**
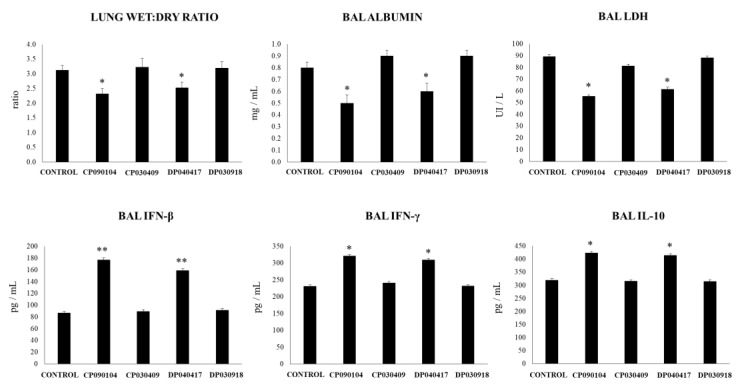
Effect of respiratory commensal bacteria strains on lung tissue damage and respiratory cytokines induced by the nasal administration of a mixture of the MALP2 and Pam3CSK4 ligands that signal through TLR2/TLR6 and TLR2/TLR1, respectively. Infant mice were nasally primed with viable *Corynebacterium pseudodiphteriticum* (CP) strains 090104 or 030409 or *Dolosigranulum pigrum* (DP) strains 040417 or 030918 during five consecutive days and then challenged with two once-daily doses of MALP2/Pam3CSK4. Non-treated infant mice challenged with MALP2/Pam3CSK4 were used as controls. Two days after the last MALP2/Pam3CSK4 administration lung wet:dry weight ratio, lactate dehydrogenase (LDH) activity and, albumin concentrations and the levels of interferon (IFN)-β, IFN-γ, and interleukin (IL)-10 in broncho-alveolar lavages (BAL) were determined. Experiments were performed with 5–6 mice per group. The results represent data from three independent experiments. Significantly different when compared to control * *p* < 0.05 or ** *p* < 0.01.

In a second set of experiments, we evaluated the capacity of the respiratory commensal bacteria strains to modulate the lung inflammatory response triggered by TLR2 activation. A mixture of the MALP2 and Pam3CSK4 ligands that signal through TLR2/TLR6 and TLR2/TLR1, respectively [27], was used to induce a TLR2-dependent inflammatory response in mice lungs. As shown in Figure 2, the nasal challenge of mice with MALP2/Pam3CSK4 increased the values of lung wet:dry ratio, BAL albumin and LDH as well as BAL IFN-β, IFN-γ and IL-10. However, the levels of lung injury markers and BAL IFN-β were significantly lower to the observed after TLR3 activation (Figure 1). On the contrary, MALP2/Pam3CSK4 stimulation was able to induce higher levels of BAL IFN-γ when compared to poly(I:C) while the levels of BAL IL-10 were similar after TLR2 and TLR3 activation. Mice nasally treated with *C. pseudodiphteriticum* 090104 or *D. pigrum* 040417 had values of lung injury markers that were significantly lower than those observed in the control group while their levels of IFN-β, IFN-γ and IL-10 in the respiratory tract were higher than controls (Figure 2). In addition, mice nasally treated with 090104 or 040417 strains had values of BAL TNF-α and neutrophils that were significantly higher and lower than controls, respectively (Figure S2C). On the contrary, mice nasally treated with *C. pseudodiphteriticum* 030409 or *D. pigrum* 030918 showed values of lung injury markers as well as BAL cytokines that were not different from the control group after stimulation with MALP2/Pam3CSK4 (Figure 2, Figure S2C).

These results indicate that the ability of respiratory commensal bacteria to modulate the respiratory innate immune response triggered by TLR2 or TLR3 is a strain-dependent property. Then, we selected *D. pigrum* 040417 for further characterization of its immunomodulatory capacities and its ability to protect against respiratory infections.

### 3.2. Dolosigranulum Pigrum 040417 Modulates Respiratory and Systemic Cytokine Profiles 

**Figure 3:**
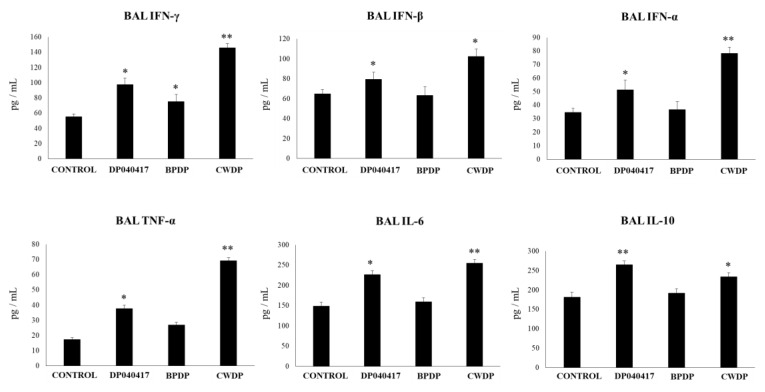
Effect of *Dolosigranulum pigrum* 040417 on respiratory cytokines. Viable (DP), bacterium-like particles (BPDP) or cell wall (CWDP) from *D. pigrum* 040417 were nasally administered to infant mice during five consecutive days. Non-treated infant mice were used as controls. Levels of tumor necrosis factor (TNF)-α, interferon (IFN)-α, IFN-β, IFN-γ, interleukin (IL)-6, and IL-10 were determined in broncho-alveolar lavages (BAL) one day after the last treatment. Experiments were performed with 5–6 mice per group. The results represent data from three independent experiments. Significantly different when compared to control * *p* < 0.05 or ** *p* < 0.01.

**Figure 4:**
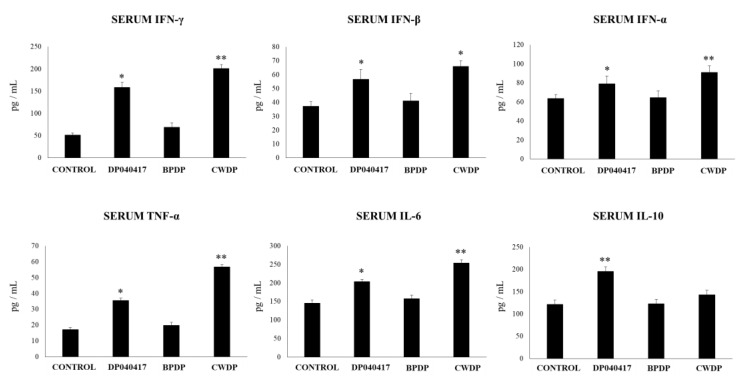
Effect of Dolosigranulum pigrum 040417 on blood cytokines. Viable (DP), bacterium-like particles (BPDP), or cell wall (CWDP) from D. pigrum 040417 were nasally administered to infant mice during five consecutive days. Non-treated infant mice were used as controls. Levels of tumor necrosis factor (TNF)-α, interferon (IFN)-α, IFN-β, IFN-γ, interleukin (IL)-6, and IL-10 were determined in serum one day after the last treatment. Experiments were performed with 5–6 mice per group. The results represent data from three independent experiments. Significantly different when compared to control * *p* < 0.05 or ** *p* < 0.01.

We reported previously that non-viable immunomodulatory bacteria or their cellular fractions were able to modulate the immunity in the respiratory tract [19,23,26]. Then, in order to further characterize *D. pigrum* 040417, we obtained BLPs (BPDP group) and its cell wall (CWDP group) and evaluated their ability to modulate respiratory and systemic immunity. *D. pigrum* 040417, BPDP or CWDP were nasally administered to infant mice for 5 consecutive days, and the levels of IFN-α, IFN-β, IFN-γ, TNF-α, IL-10, and IL-6 were quantified in BAL (Figure 3) and serum (Figure 4) samples. *D. pigrum*- and CWDP-treated mice showed a significant increase in the levels of BAL TNF-α, IL-6, IFN-γ and IFN-α when compared to control mice. CWDP was more efficient than viable bacteria to improve the levels of respiratory TNF-α, IFN-γ and IFN-α (Figure 3). BAL IFN-β levels were only augmented by the CWDP treatment. The immunoregulatory cytokine IL-10 in the respiratory tract was also detected in higher concentrations in mice treated with *D. pigrum* 040417 and CWDP when compared to the control group, although viable bacteria were more efficient to improve the levels of this cytokine (Figure 3). No differences in the numbers of BAL macrophages or neutrophils were found when treated mice were compared to controls (Figure S3).

When serum cytokines were evaluated, it was observed that the levels of TNF-α, IFN-β, IFN-γ, and IL-6 were significantly higher in infant mice treated with *D. pigrum* 040417 or CWDP when compared to control mice (Figure 4). Serum TNF-α was higher in CWDP-treated mice than in those receiving *D. pigrum* 040417. The levels of IFN-α in blood were not affected by any of the treatments while only *D. pigrum* 040417 increased serum IL-10 (Figure 4).

### 3.3. Dolosigranulum Pigrum 040417 Differentially Modulates Lung Immune Cells

**Figure 5:**
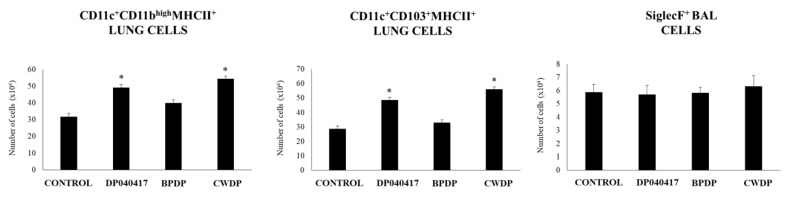
Effect of *Dolosigranulum pigrum* 040417 on respiratory immune cell populations. Viable (DP), bacterium-like particles (BPDP) or cell wall (CWDP) from *D. pigrum* 040417 were nasally administered to infant mice during five consecutive days. Non-treated infant mice were used as controls. The numbers of MHC-II^+^CD11c^+^CD11b^low^CD103^+^ and MHC-II^+^CD11c^+^CD11b^high^CD103^-^ dendritic cells in lungs, and CD45^+^MHC-II^-^CD11c^+^SiglecF^+^ alveolar macrophages in broncho-alveolar lavages (BAL) were determined by flow cytometry one day after the last treatment. Experiments were performed with 5–6 mice per group. The results represent data from three independent experiments. Significantly different when compared to control * *p* < 0.05 or ** *p* < 0.01

We next evaluated the influence of the nasal administration of *D. pigrum* 040417, BPDP or CWDP on lung immune cell populations by using flow cytometry. The numbers of lung CD11c^+^CD11b^high^MHCII^+^ and lung CD11c^+^CD103^+^MHCII^+^ cell populations were significantly increased in *D. pigrum* 040417- and CWDP-treated mice when compared to controls (Figure 5, Figure S4). The numbers of BAL SiglecF^+^ cells were not modified by any of the treatments (Figure 5). 

**Figure 6:**
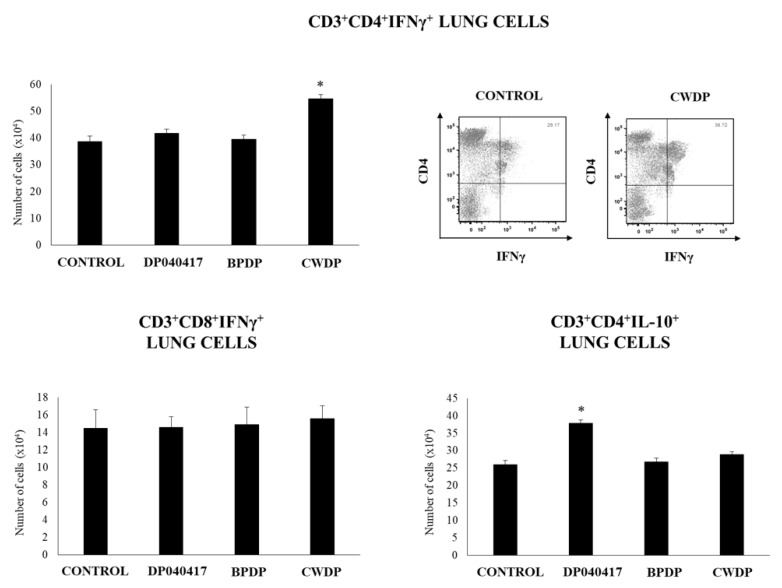
Effect of *Dolosigranulum pigrum* 040417 on respiratory immune cell populations. Viable (DP), bacterium-like particles (BPDP) or cell wall (CWDP) from *D. pigrum* 040417 were nasally administered to infant mice during five consecutive days. Non-treated infant mice were used as controls. The numbers of CD3^+^CD4^+^IFN-γ^+^, CD3^+^CD4^+^IL-10^+^, and CD3^+^CD8^+^IFN-γ^+^ T lymphocytes in lungs were determined by flow cytometry one day after the last treatment. Experiments were performed with 5–6 mice per group. The results represent data from three independent experiments. Significantly different when compared to control * *p* < 0.05 or ** *p* < 0.01.

We also evaluated T cell populations in lungs (Figure 6, Figure S5). No differences were found between treated and control mice when the levels of lung CD3^+^CD4^+^ and CD3^+^CD8^+^ T cells were compared (Figure S5). In addition, the treatments were not able to modify the numbers of lung CD3^+^CD8^+^IFN-γ^+^ T cells. However, CWDP-treated mice showed enhanced numbers of lung CD3^+^CD4^+^IFN-γ^+^ T cells than control mice. In addition, *D. pigrum* 040417 increased lung CD3^+^CD4^+^IL-10^+^ T cells when compared to the control group (Figure 6).

### 3.4. Dolosigranulum Pigrum 040417 Diminish the TLR3-Mediated Inflammatory Lung Damage

**Figure 7:**
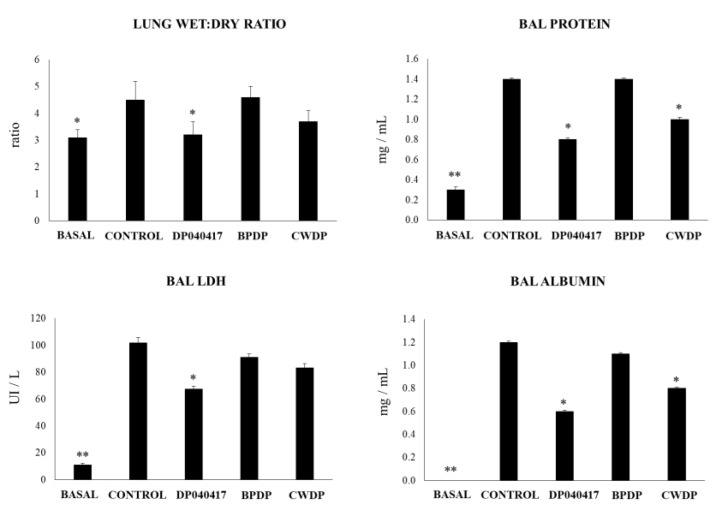
Effect of *Dolosigranulum pigrum* 040417 on lung tissue damage induced by the nasal administration of the viral pathogen-associated molecular pattern poly(I:C). Infant mice were nasally primed with viable (DP), bacterium-like particles (BPDP) or cell wall (CWDP) from *D. pigrum* 040417 during five consecutive days and then challenged with three once-daily doses of poly(I:C). Non-treated infant mice challenged with poly(I:C) were used as controls. Two days after the last poly(I:C) administration lung wet:dry weight ratio, lactate dehydrogenase (LDH) activity and, albumin and protein concentrations in broncho-alveolar lavages (BAL) were determined. Experiments were performed with 5–6 mice per group. The results represent data from three independent experiments. Significantly different when compared to control * *p* < 0.05 or ** *p* < 0.01.

The impact of BPDP or CWDP on the TLR3-triggered respiratory response were evaluated and compared with the effect of *D. pigrum* 040417. As described before, nasal administration of the viral nucleic acid analogue and TLR3 agonist poly(I:C) to infant mice significantly increased the levels of lung injury markers (Figure 7). Similar to *D. pigrum* 040417, the nasal priming with CWDP significantly reduced lung wet:dry ratio, and the levels of BAL proteins and albumin when compared to poly(I:C)-challenged controls. Only the *D. pigrum* 040417 was able to reduce the levels of BAL LDH while the mice treated with BPDP showed levels of lung injury markers that were not different from control mice (Figure 7).

**Figure 8:**
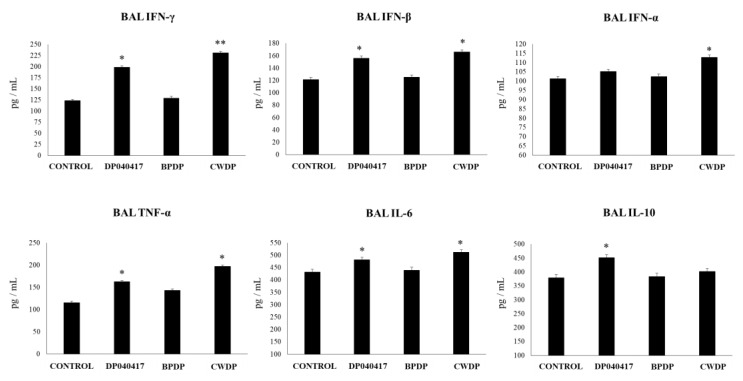
Effect of *Dolosigranulum pigrum* 040417 on respiratory cytokines after the nasal administration of the viral pathogen-associated molecular pattern poly(I:C). Infant mice were nasally primed with viable (DP), bacterium-like particles (BPDP) or cell wall (CWDP) from *D. pigrum* 040417 during five consecutive days and then challenged with three once-daily doses of poly(I:C). Non-treated infant mice challenged with poly(I:C) were used as controls. Two days after the last poly(I:C) administration the levels of tumor necrosis factor (TNF)-α, interferon (IFN)-α, IFN-β, IFN-γ, interleukin (IL)-6, and IL-10 were determined in broncho-alveolar lavages (BAL). Experiments were performed with 5–6 mice per group. The results represent data from three independent experiments. Significantly different when compared to control * *p* < 0.05 or ** *p* < 0.01.

We further evaluated the respiratory cytokine profile in mice treated with *D. pigrum* 040417, BPDP or CWDP and challenged with poly(I:C). As shown in Figure 8, *D. pigrum* 040417 and CWDP significantly increased BAL IFN-β and IFN-γ levels when compared to controls. None of the three treatments was able to modify the concentrations of BAL IFN-α while the three *D. pigrum* 040417, BPDP and CWDP significantly increased BAL TNF-α and IL-6. Of note, mice receiving CWDP showed BAL TNF-α and IL-6 levels that were higher than the observed in *D. pigrum* 040417 and BPDP groups (Figure 8). Only the *D. pigrum* 040417 was able to increase the levels of BAL IL-10.

**Figure 9:**
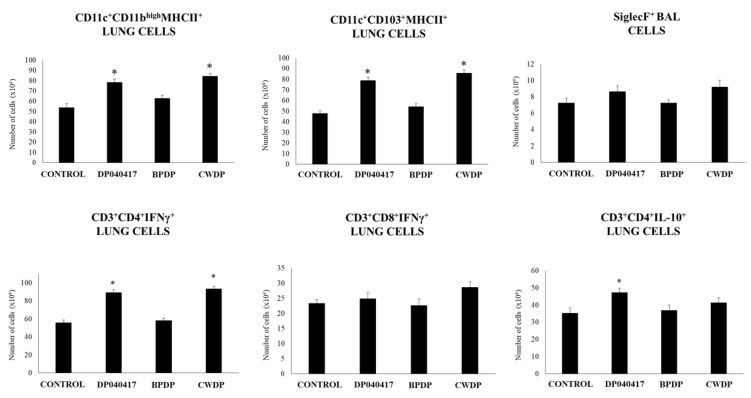
Effect of *Dolosigranulum pigrum* 040417 on respiratory immune cell populations after the nasal administration of the viral pathogen-associated molecular pattern poly(I:C). Infant mice were nasally primed with viable (DP), bacterium-like particles (BPDP) or cell wall (CWDP) from *D. pigrum* 040417 during five consecutive days and then challenged with three once-daily doses of poly(I:C). Non-treated infant mice challenged with poly(I:C) were used as controls. Two days after the last poly(I:C) administration, the numbers of lung T cells including CD3^+^CD4^+^IFN-γ^+^, CD3^+^CD4^+^IL-10^+^, and CD3^+^CD8^+^IFN-γ^+^ T lymphocytes, as well as antigen-presenting cells including MHC-II^+^CD11c^+^CD11b^low^CD103^+^ and MHC-II^+^CD11c^+^CD11b^high^CD103^-^ dendritic cells, and CD45^+^MHC-II^-^CD11c^+^SiglecF^+^ alveolar macrophages were determined by flow cytometry. Experiments were performed with 5–6 mice per group. The results represent data from three independent experiments. Significantly different when compared to control * *p* < 0.05 or ** *p* < 0.01.

The analysis of lung immune cell populations showed that both *D. pigrum* 040417 and CWDP were able to significantly increase the numbers of lung CD11c^+^CD11b^high^MHCII^+^ and CD11c^+^CD103^+^MHCII^+^ cells in mice challenged with poly(I:C) while the treatments were not able to modify the numbers of BAL SiglecF^+^ cells when compared to controls (Figure 9). BPDP treatment was not able to influence the number of antigen-presenting cell populations in the respiratory tract. In addition, it was found that both *D. pigrum* 040417 and CWDP significantly augmented the numbers of CD3^+^CD4^+^IFNγ^+^ T cells in lungs (Figure 9). The number of lung CD3^+^CD4^+^IL-10^+^ T cells was significantly higher in the group receiving *D. pigrum* 040417 compared to control. In contrast, no differences were observed in lung CD3^+^CD8^+^IFNγ^+^ T cells among the experimental groups (Figure 9).

### 3.5. Dolosigranulum Pigrum 040417 Improves Resistance to RSV Infection

**Figure 10:**
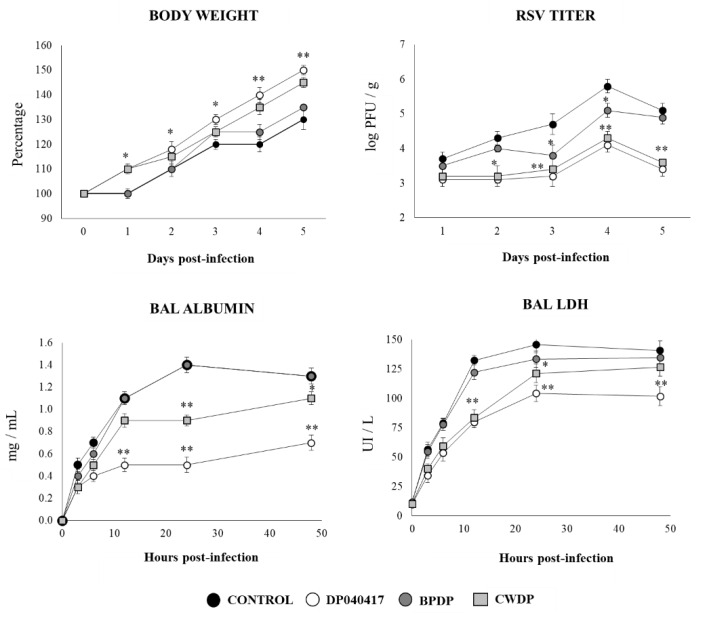
Effect of *Dolosigranulum pigrum* 040417 on the resistance to Respiratory Syncytial Virus (RSV) infection. Infant mice were nasally primed with viable (DP), bacterium-like particles (BPDP), or cell wall (CWDP) from *D. pigrum* 040417 during five consecutive days and then challenged with RSV. Non-treated infant mice challenged with the viral pathogen were used as controls. Lung RSV titers, changes in body weight, and lactate dehydrogenase (LDH) activity and albumin concentrations in broncho-alveolar lavages (BAL) were evaluated on different time points after the viral challenge. Experiments were performed with 5–6 mice per group per each time point. The results represent data from three independent experiments. Significantly different when compared to control at the same time point * *p* < 0.05 or ** *p* < 0.01.

Taking into consideration the ability of *D. pigrum* 040417 and CWDP to differentially regulate the respiratory TLR3-mediated innate immune response, we next aimed to evaluate whether these treatments were capable of increasing the resistance to a real viral challenge. Then, challenge infection experiments were performed with RSV. The BPDP treatment was also included in experiments for comparison. As shown in Figure 10, both *D. pigrum* 040417 and CWDP significantly improved the body weight gain that was affected by the RSV infection. The virus was detected in the lungs of all experimental groups during the five days studied. The RSV loads were 2 log units lower in mice treated with *D. pigrum* 040417 or CWDP than in control mice, being both treatments equally effective to reduce the RSV replication in the respiratory tract (Figure 10). In addition, the markers of lung tissue damage in RSV-infected mice showed that the viral infection induced significant cellular damage and alveolar-capillary barrier alterations. Both albumin and LDH concentrations in BAL were significantly lower in infant mice previously treated with *D. pigrum* 040417 or CWDP than in RSV-challenged controls. Although CWDP was also able to reduce the damage marker levels in BAL, DP was the most effective treatment (Figure 10). Body weight gain, lung RSV titers and BAL injury markers in BPDP-treated infant mice were not different from the control group.

### 3.6. Respiratory Commensal Bacteria Improve Resistance to Pneumococcal Infection

**Figure 11:**
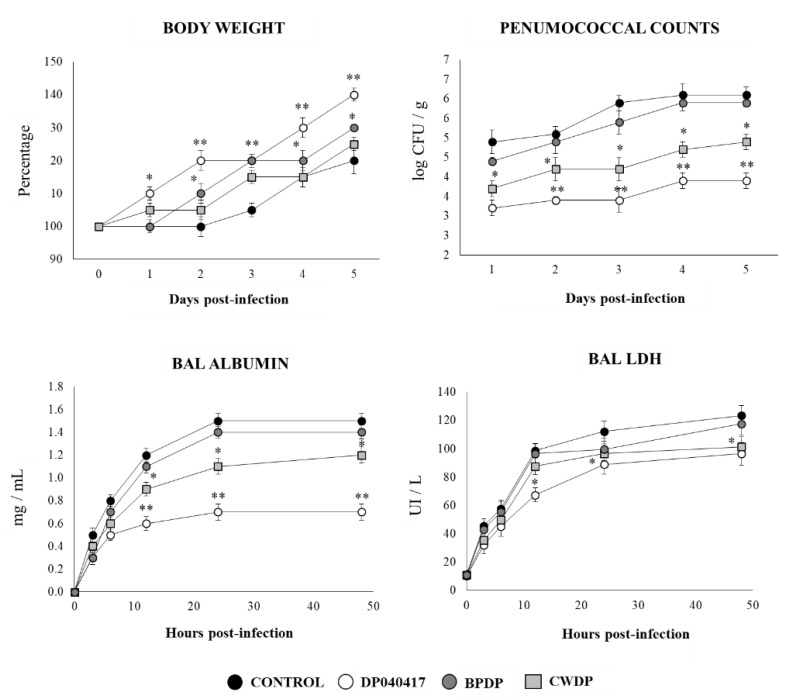
Effect of *Dolosigranulum pigrum* 040417 on the resistance to *Streptococcus pneumoniae* infection. Infant mice were nasally primed with viable (DP), bacterium-like particles (BPDP) or cell wall (CWDP) from *D. pigrum* 040417 during five consecutive days and then challenged with pneumococci. Non-treated infant mice challenged with the bacterial pathogen were used as controls. Lung pneumococcal cell counts, changes in body weight, and lactate dehydrogenase (LDH) activity and albumin concentrations in broncho-alveolar lavages (BAL) were evaluated on different time points after the viral challenge. Experiments were performed with 5–6 mice per group per each time point. The results represent data from three independent experiments. Significantly different when compared to control at the same time point * *p* < 0.05 or ** *p* < 0.01.

Finally, we aimed to evaluate whether *D. pigrum* 040417, BPDP or CWDP were able to influence the resistance of infant mice to the primary infection with *S. pneumoniae* (Figure 11). Similar to RSV infection, the challenge of infant mice with *S. pneumoniae* induced a delay in the body weight gain of control mice. The infant mice treated with *D. pigrum* 040417 showed improved body weight gain after the pneumococcal infection. In addition, *D. pigrum* 040417-treated mice had significantly lower pneumococcal cell counts in lungs as well as reduced levels of albumin and LDH concentrations in BAL when compared to controls (Figure 11). The CWDP treatment was able to reduce pneumococci counts and the levels of the markers of lung tissue damage; however, its effect was significantly lower when compared to *D. pigrum* 040417. On the contrary, BPDP induced no beneficial effect on pneumococcal infection.

**Figure 12:**
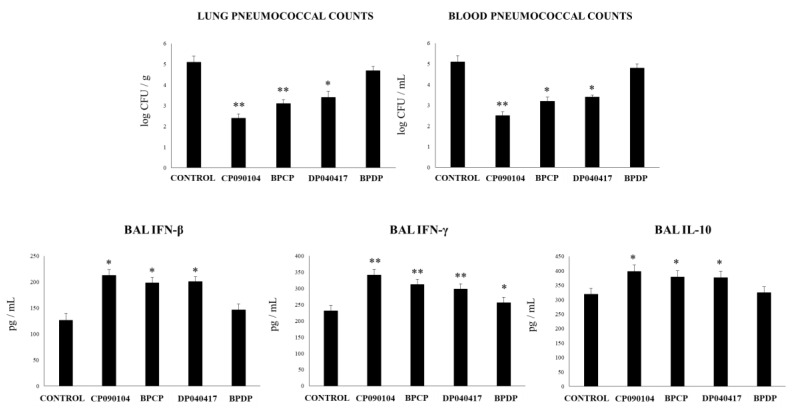
Effect of *Dolosigranulum pigrum* 040417 and *Corynebacterium pseudodiphteriticum* 090104 on the resistance and immune response to *Streptococcus pneumoniae* infection. Infant mice were nasally primed with viable (DP) or bacterium-like particles (BPDP) from *D. pigrum* 040417, or viable (CP) or bacterium-like particles (BPCP) from *C. pseudodiphteriticum* 090104 during five consecutive days and then challenged with pneumococci. Non-treated infant mice challenged with the bacterial pathogen were used as controls. Lung and blood pneumococcal cell counts and the levels of interferon (IFN)-β, IFN-γ, and interleukin (IL)-10 were determined in broncho-alveolar lavages (BAL) on day two post-infection. Experiments were performed with 5–6 mice per group per each time point. The results represent data from three independent experiments. Significantly different when compared to control * *p* < 0.05 and ** *p* < 0.01.

We previously reported that *C. pseudodiphteriticum* 090104 was able to increase the resistance of infant mice to secondary pneumococcal pneumonia [19]. However, its effect on primary pneumococcal infection was not assessed in depth. Moreover, the ability of BLPs obtained from *C. pseudodiphteriticum* 090104 in this context was not evaluated before. Then, we performed experiments to compare the influence of *D. pigrum* 040417 and *C. pseudodiphteriticum* 090104 as well as their derived BLPs on the resistance and the innate immune response to pneumococcal infection. Similar to *D. pigrum* 040417, the treatment with *C. pseudodiphteriticum* 090104 significantly reduced the pneumococcal cell counts in lung and blood when compared to controls (Figure 12). BLPs from *C. pseudodiphteriticum* 090104 were as efficient as viable commensal respiratory bacteria to diminish *S. pneumoniae* counts in lung and blood while the BLPs from *D. pigrum* 040417 induced no significant effect when compared to controls. *D. pigrum* 040417, *C. pseudodiphteriticum* 090104 and the BLPs from the 090104 strain significantly increased the levels of BAL IFN-β, IFN-γ or IL-10 when compared to controls (Figure 12). BLPs from *D. pigrum* 040417 induced no significant effects on BAL IFN-β or IL-10 when compared to control mice. In addition, this treatment increased the levels of BAL IFN-γ; however, the values were significantly lower than the observed in *D. pigrum* 040417, *C. pseudodiphteriticum* 090104 or BLPs from the 090104 strain groups (Figure 12).

## 4. Discussion

The great advances in sequencing techniques have significantly improved our understanding of the composition of the respiratory microbiota as well as its functional properties [27]. Considering that the nose and nasopharynx are of easy access and that most of the pathogenic microorganisms that cause respiratory and invasive infections permanently or temporarily colonize these mucosal tissues, the study of the respiratory microbiota has focused mainly in those niches. In this regard, large-scale sequencing studies characterizing the microbiota of the upper respiratory tract of children [28,29,30] and adults [14] have found that the nose and the nasopharynx microbial populations are dominated by an intermixed profile where Staphylococcus, Corynebacterium, and Dolosigranulum are important bacterial members. Moreover, Corynebacterium and Dolosigranulum species are now considered beneficial members of the upper respiratory microbiota because of their ability to induce protection against bacterial [15,16,17] and viral [18,19] respiratory pathogens. In this work, we have advanced in the characterization of the beneficial properties of *C. pseudodiphtheriticum* and *D. pigrum* strains by evaluating their ability to modulate the respiratory innate immunity and the resistance against bacterial and viral pathogens. Four important conclusions can be inferred from the analysis of the results presented in this work. 

### 4.1. The Ability of Respiratory Commensal Bacteria to Modulate the Innate Immunity in the Respiratory Tract is a Strain-Dependent Characteristic 

Our results demonstrated for the first time the strain-dependent ability of *C. pseudodiphtheriticum* and *D. pigrum* to modulate respiratory immunity.

Decades of research dedicated to the evaluation of traditional probiotic microorganisms (mainly from the *Lactobacillus* and *Bifidobacterium* species) have made it possible to define a series of characteristics that a certain microorganism must gather to be considered a probiotic [8]. The most important property for a probiotic candidate is to provide a benefit to the host that is scientifically proven. Indeed, the most used probiotic strain globally have undergone rigorous in vitro and in vivo studies as well as clinical trials in order to conclusively demonstrate their beneficial effects. Another important conclusion after years of probiotic research is that their beneficial effects are strain-specific, and therefore, the activity of one strain cannot be extrapolated to others even of the same species [20]. Hence, in order to propose the use of respiratory commensal bacteria strains as next generation probiotics, high-quality scientific evidence-based studies are mandatory. Moreover, to determine whether the beneficial effects of respiratory commensal bacteria is a strain-dependent characteristic is also of high importance in order to select the most efficient candidates. 

To evaluate the effect of different *C. pseudodiphtheriticum* and *D. pigrum* strains on respiratory immunity, we used two models of TLR-triggered respiratory innate immune responses. On the one hand, considering that lungs have a high expression of TLR2 [31] and that this signaling pathway is activated by Gram positive pathogens [27], a mixture of the MALP2 and Pam3CSK4 ligands was used to mimic the respiratory pro-inflammatory response induced Gram positive bacterial infections. MALP-2 activate TLR2/TLR6 heterodimers using CD36 as a co-receptor [32] and strongly stimulates the production of pro-inflammatory cytokines and chemokines in the respiratory tract inducing the infiltration and activation of immune cells [33,34]. Pam3CSK4 acts on TLR1/TLR2 heterodimers [27], and in vitro studies in bronchial epithelial cells and alveolar macrophages as well as in vivo studies in mice have demonstrated its ability to strongly augment the production of TNF-α, IL-1β, IL-6, MCP-1 and IL-8 by respiratory cells [35,36,37]. Interestingly, it was reported that in TLR6-deficient mice the production of TNF-α, IL-1β, and IL-6 in response to challenges with TLR2 ligands is not altered, but that levels of IFN-γ and IL-10 are significantly reduced, indicating that those cytokines are regulated mainly by TLR2/TLR6-receptor heterodimers [38]. Therefore, considering our previous results evaluating the effects of immunobiotics on respiratory immunity that demonstrated their strong influence in the expression of pro-inflammatory cytokines as well as IFN-γ and IL-10 [19,23,25,26], we decided to use a mixture MALP2 and Pam3CSK4 to evaluate the effect of respiratory commensal bacteria strains. On the other hand, to mimic the respiratory inflammatory response induced by viral infections, we used the TLR3 ligand poly(I:C). As it has been described by us and others, nasally administered poly(I:C) primes TLR3 and induces a marked inflammatory damage characterized by impaired alveolar–capillary barrier function and epithelial cell death as well as increased levels of TNF-α, IL-1β, IL-6, MCP-1, IL-8, IFN-β, and IFN-γ [25,26].

The results of this work demonstrated that nasally administered *C. pseudodiphteriticum* 090104 or *D. pigrum* 040417 were able to improve the levels IFN-β, TNF-α, and IFN-γ and increase IL-10 in the respiratory tract as well as significantly diminish the markers of lung tissue damage after the activation of TLR2 or TLR3 in the respiratory tract. Of note, the treatments with *C. pseudodiphteriticum* 030409 or *D. pigrum* 030918 were not able to modify the levels of cytokines or reduce the lung damage when compared to controls, demonstrating a clear strain-dependent immunomodulatory effect of respiratory commensal bacteria.

### 4.2. D. pigrum 040417, Similar to C. Pseudodiphteriticum 090104, Is Capable of Modulating the Respiratory Innate Immunity and Improve the Resistance to RSV and S. Pneumoniae Infections 

We previously reported that the nasal administration of *C. pseudodiphtheriticum* 090104 to mice diminished pneumococcal cell counts in lungs and avoided its blood dissemination after the primary infection with RSV [19]. In this work, we confirmed that the 040417 strain is capable to improve the resistance of mice to primary pneumococcal infection, an effect that is shared with *D. pigrum* 040417. We also demonstrated that both immunobiotic respiratory commensal bacteria were able to reduce the lung titers of RSV and diminish the lung tissue damage triggered by the viral infection. Those beneficial effects were related to the ability of respiratory commensal bacteria to enhance the activities of antigen-presenting cells and T cells. Both, *C. pseudodiphtheriticum* 090104 [19] and *D. pigrum* 040417 were able to increase the numbers of lung CD11c^+^CD11b^high^MHCII^+^ and CD11c^+^CD103^+^MHCII^+^ DCs as well as the numbers of lung CD3^+^CD4^+^IFN-γ^+^ T cells, indicating their ability to stimulate Th1 responses. 

Appropriate production of IFN-γ in the respiratory tract has been associated with the protection against RSV and *S. pneumoniae*. Improved levels of IFN-γ stimulates pulmonary macrophages and DCs that are critical for host defense against pneumococcal infection [39]. Genome-wide microarray-based transcriptional analysis of whole lungs of mice infected with *S. pneumoniae* revealed that the up-regulation of IFN-γ and IFN-γ-related genes was associated with the protection against this respiratory pathogen [40]. On the other hand, IFN-γ is essential to mounting an adequate immune response during RSV infections in mice and humans. The IFN-γ-mediated activation of alveolar macrophages is essential for efficient viral clearance [41]. It was suggested that quantitative and qualitative differences in IFN-γ production could explain the different susceptibility of infant and adult mice to RSV infection [42]. While adult mice accumulate both CD8^+^CD44^high^IFN-γ^+^ and CD4^+^CD44^high^IFN-γ^+^ T in the alveolar space after the challenge with RSV, infant mice accumulate mainly CD8^+^CD44^high^IFN-γ^+^ T cells in lung tissue and airway. Then, the lack of effective recruitment of CD4^+^IFN-γ^+^ T cells into the alveolar space and the lower activation of macrophages correlated with the higher ability of RSV to replicate in infant lungs. These previous findings and the results of this work highlight the role of CD4^+^IFN-γ^+^ T cells in the beneficial effects induced by immunobiotic respiratory commensal bacteria. Moreover, our results open the possibility of further research in the future regarding the potential beneficial effect of *C. pseudodiphtheriticum* 090104 or *D. pigrum* 040417 in the protection of neonatal mice against RSV infection. In line with this hypothesis, it was demonstrated that the nasal priming of neonatal mice with the *Enterococcus faecalis* CNCM I 4969 strain, which was originally isolated from mouse neonatal lungs, significantly stimulated respiratory Th1 responses and beneficially influenced the outcome of allergic asthma development [43]. 

Our results also showed that 090104 and 040417 strains increased lung CD3^+^CD4^+^IL-10^+^ T cells. The regulation of excessive inflammation is a key factor in the outcome of both RSV [44] and *S. pneumoniae* [45,46] infections. However, IL-10 has been associated with both beneficial and detrimental effects during respiratory infections. While IL-10 increase is necessary for limiting the actions of pro-inflammatory cytokines and cells, and reducing the inflammatory-mediated tissue damage that compromises the lung function, delayed and/or over-expressed IL-10 impairs the immune mechanisms involved in the control of respiratory pathogens growth [47,48]. Our results allowed us to speculate that the increases in respiratory IL-10 induced by immunobiotic commensal respiratory bacteria would be associated with the regulation of lung inflammatory cell infiltration and the protection against tissue damage; however, further detailed kinetics studies would be of value to precisely establish the role of CD3^+^CD4^+^IL-10^+^ T cells and Il-10 in the improved resistance against infections. 

Of note, although *C. pseudodiphtheriticum* 090104 or *D. pigrum* 040417 were able to increase the resistance to RSV and *S. pneumoniae* infections none of the immunobiotic commensal bacteria managed to completely avoid the infections with the respiratory pathogens. It was shown that the members of the respiratory microbiota are able to modulate each other’s growth by direct and indirect mechanisms. Interestingly, the frequent co-occurrence of *Corynebacterium* spp. and *Dolosigranulum* spp. in the nasopharynx has been attributed to the ability of *Dolosigranulum* spp. to acidificate the local environment, facilitating the expansion of *Corynebacterium* spp. [15,49,50]. Then, to evaluate whether the nasal administration of an “immunobiotic consortium” constituted by *C. pseudodiphtheriticum* 090104 or *D. pigrum* 040417 is more efficient that individual strains to modulate respiratory immunity is also an interesting topic for research. 

### 4.3. The Ability of BLP Derived from Respiratory Commensal Bacteria to Modulate the Innate Immunity in the Respiratory Tract is also a Strain Dependent Characteristic

Our previous studies comparing the effect of viable and non-viable *C. pseudodiphtheriticum* 090104 in the modulation of respiratory immunity demonstrated that the viability, and probably the colonization of the respiratory mucosa, are necessary for the optimal immunomodulatory effect [19]. Our comparative experiments performed with *C. pseudodiphtheriticum* 090104 and its BLP demonstrated that BLP from 090104 strain were not able to improve the production of IFN-β by CD45^+^SiglecF^+^ alveolar macrophages or IL-10 by CD4^+^ T cells. Moreover, although BLP from *C. pseudodiphtheriticum* 090104 increased the numbers of lung CD4^+^ IFN-γ^+^ T cells and the levels of BAL IFN-γ, both parameters were significantly lower when compared with the viable bacteria [19]. The comparative experiments performed in this work with viable and non-viable *D. pigrum* 040417 confirmed the necessity of viability in achieving the optimal immunomodulatory effect. However, BLPs from *D. pigrum* 040417 were not able to modulate the respiratory immunity or enhance protection against *S. pneumoniae* or RSV. BLPs from *D. pigrum* 040417 did not modulate the respiratory levels of IFN-γ or IL-10 that are known to be involved in the protection against respiratory pathogens´ growth and inflammatory-mediated lung damage, respectively, as described above. The BLPs from the 040417 strain were also incapable of increasing respiratory levels of IFN-β. The improved levels of IFN-β induced by immunobiotic bacteria have been associated with the increased expression of antiviral factors that limit viral replication in the respiratory tract [26,51]. In addition, IFN-β has been associated with the control of pneumococcal dissemination into the blood since pneumococci were observed earlier and at higher numbers in blood samples of IFNAR1^−/−^ mice compared to wild type animals, while the nasal administration of IFN-β increased the protection of mice against pneumococcal systemic disease [52].

Therefore, the results of the present study suggest that is not possible to predict an immunomodulatory effect for a BLP by evaluating the viable bacteria. BLPs derived from immunomodulatory bacteria should be carefully studied to find out if they retain the immunomodulatory properties and if they do, whether or not the effect is similar to that induced by viable bacteria. These findings are in line with our recent study reporting that BLPs from distinct immunomodulatory lactobacilli differs in their capacity to modulate the intestinal immunity [21].

### 4.4. The Cell Wall of D. pigrum 040417 is an Interesting Alternative to Beneficially Modulate the Respiratory Innate Immune Response in High-Risk Populations

Safety is an important property for a probiotic microorganism. Traditional *Lactobacillus* spp. and *Bifidobacterium* spp. probiotic strains are included in the group of Generally Regarded as Safe (GRAS) microorganisms because of their proved safety [8]. On the contrary, the new species of potential beneficial commensal bacteria isolated from intestinal or respiratory tissues have not been evaluated in depth regarding their beneficial or their potential detrimental effects. There are some reports indicating opportunistic infections caused by *D. pigrum*. This species has been associated with non-ventilator-associated nosocomial pneumonia and septicemia [53] as well as to ventilator-associated pneumonia [54]. In addition, this bacterium has been implicated as an agent of lung infection in cystic fibrosis patients [55]. It should be noted that in all the reported cases of *D. pigrum*-associated respiratory infection, no young or adult immunocompetent hosts were described. On the contrary, most of the cases involved elderly suffering other medical conditions such as chronic respiratory insufficiency. Although some safety concerns about this bacterium may arise, especially in immunocompromised populations, our studies indicate that the use of *D. pigrum* 040417 was safe in the mouse model used here. More genetic and genomic studies aimed to characterize the potentially dangerous genes in this bacterium, as well as studies in animal models of high-risk populations are necessary to definitely ensure the safety of this *D. pigrum* strain.

The use of cellular components instead of viable bacteria could offer opportunities for immunomodulation in high-risk populations in which the use of live microorganisms could represent a potential danger [20,26,56]. In this regard, we have previously reported that the nasal priming with peptidoglycan from the immunobiotic *Lactobacillus rhamnosus* CRL1505 enhance the resistance of infant mice to primary RSV infection and secondary pneumococcal pneumonia [26]. In addition, we demonstrated that the cell wall and the peptidoglycan of *L. rhamnosus* CRL1505 increased the resistance of immunocompromised malnourished mice to primary pneumococcal infection [20,56]. Surprisingly, although BLPs from *D. pigrum* 040417 did not modulate respiratory immunity, its purified cell wall (CWDP) was able to increase lung CD11c^+^CD11b^high^MHCII^+^ and CD11c^+^CD103^+^MHCII^+^ DCs, and CD3^+^CD4^+^IFN-γ^+^ T cells as well as the respiratory levels of IFN-γ and IFN-β. The immunomodulatory effect of CWDP correlated with an improved response against both RSV and *S. pneumoniae*. Of note, CWDP was not capable of enhancing IL-10 levels or lung CD3^+^CD4^+^IL-10^+^ T cells, which was in line with their lower ability to reduce lung damage parameters during bacterial and viral infections when compared to viable *D. pigrum* 040417. Further studies are necessary to explain the differences in the immunomodulatory abilities of BLPs and the cell wall from *D. pigrum* 040417. A possible explanation is that in obtaining the cell wall, the fragments that are recognized by the pattern recognition receptors expressed in the respiratory mucosa are more exposed, resulting in an improved activation of the innate immune system.

## 5. Conclusions

In this work, we demonstrated that respiratory commensal bacteria exert health benefits by modulating host immune responses in the respiratory tract, providing a better resistance to bacterial and viral infection. We reported for the first time that the immunomodulatory effects of respiratory commensal bacteria are a strain-dependent characteristic. Our study is a step forward in the positioning of certain strains of respiratory commensal bacteria as next-generation probiotics for the respiratory tract.

## Supplementary Materials

The following are available online at https://www.mdpi.com/2076-2607/8/5/727/s1, Figure S1: Effect of respiratory commensal bacteria strains on lung tissue damage and respiratory cytokines, Figure S2: Effect of respiratory commensal bacteria strains on broncho-alveolar lavages leucocytes and TNF-α, Figure S3: Effect of *Dolosigranulum pigrum* 040417 on broncho-alveolar lavages immune cell populations, Figure S4: Effect of *Dolosigranulum pigrum* 040417 on respiratory dendritic cell populations, Figure S5: Effect of *Dolosigranulum pigrum* 040417 on respiratory T cell populations.
