# Supplementary material for: The Ability of Respiratory Commensal Bacteria to Beneficially Modulate the Lung Innate Immune Response Is a Strain Dependent Characteristic
Source: Microorganisms. 2020 May 13;8(5):727. doi: 10.3390/microorganisms8050727 (PMC7285514; doi:10.3390/microorganisms8050727)
Supplement: Supplementary file 1 [file microorganisms-08-00727-s001.pdf]

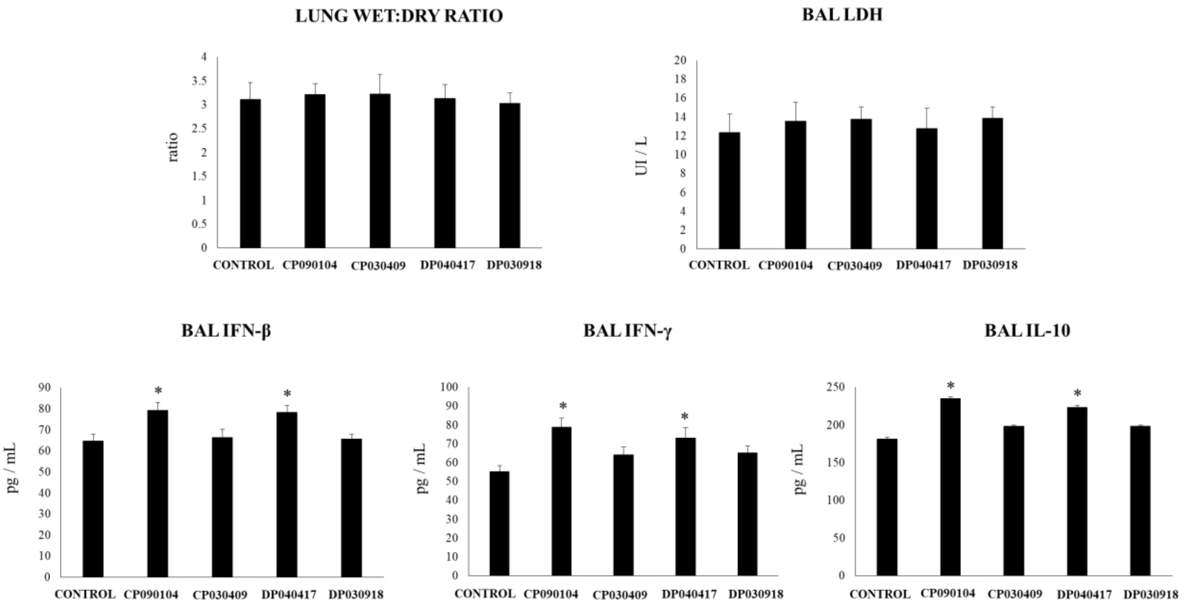

Supplementary Figure 1. Effect of respiratory commensal bacteria strains on lung tissue damage and respiratory cytokines. Infant mice were nasally primed with viable *Corynebacterium pseudodiphteriticum* (CP) strains 010904 or 030409 or *Dolosigranulum pigrum* (DP) strains 040417 or 030918 during five. Non-treated infant mice were used as controls. One day after the last bacterial administration lung wet:dry weight ratio, lactate dehydrogenase (LDH) activity and the levels of interferon (IFN)-β, IFN-γ, and interleukin (IL)-10 in broncho-alveolar lavages (BAL) were determined. Experiments were performed with 5–6 mice per group. The results represent data from three independent experiments. Significantly different when compared to control * $p < 0.05$ or ** $p < 0.01$.

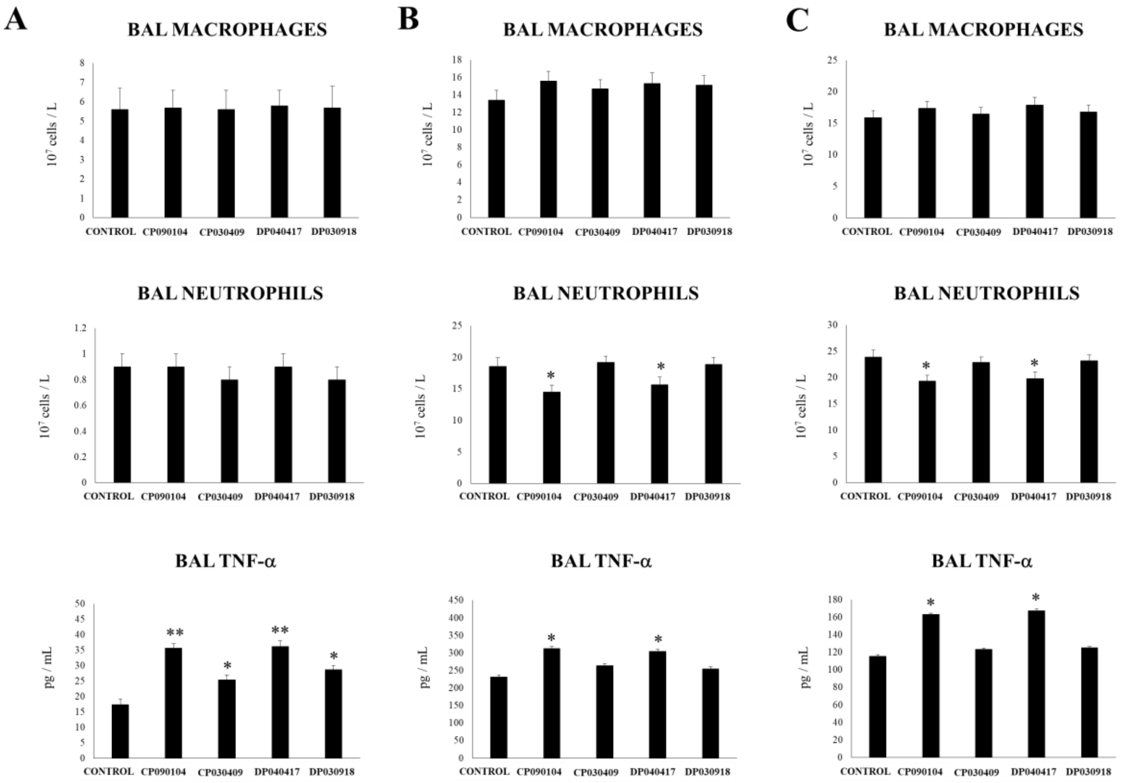

Supplementary Figure 2. Effect of respiratory commensal bacteria strains on broncho-alveolar lavages (BAL) leucocytes and TNF-α in basal conditions (**A**), or after the nasal administration of a mixture of the MALP2 and Pam3CSK4 (**B**) or poly(I:C) (**C**). Infant mice were nasally primed with viable *Corynebacterium pseudodiphteriticum* (CP) strains 010904 or 030409 or *Dolosigranulum pigrum* (DP) strains 040417 or 030918 during five consecutive days and then challenged with two once-daily doses of MALP2/Pam3CSK4 or three daily doses of poly(I:C). Non-bacterial treated infant mice were used as controls. One day after bacterial administration (A) or two days after the last MALP2/Pam3CSK4 (B) or poly(I:C) (C) administration BAL macrophages, neutrophils and TNF-α were determined. Experiments were performed with 5–6 mice per group. The results represent data from three independent experiments. Significantly different when compared to control * $p < 0.05$ or ** $p < 0.01$.

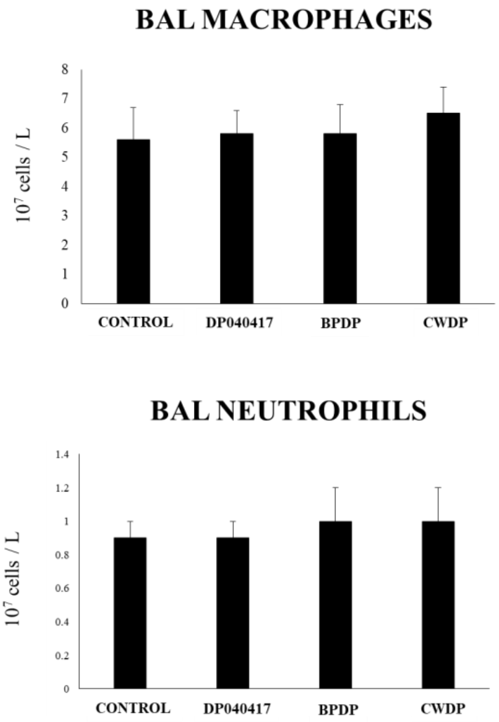

Supplementary Figure 3. Effect of *Dolosigranulum pigrum* 040417 on broncho-alveolar lavages (BAL) immune cell populations. Viable (DP), bacterium-like particles (BPDP) or cell wall (CWDP) from *D. pigrum* 040417 were nasally administered to infant mice during five consecutive days. Non-treated infant mice were used as controls. One day after the last bacterial administration BAL macrophages and neutrophils were determined. Experiments were performed with 5–6 mice per group. The results represent data from three independent experiments.

LUNG CELLS DENDRITIC CELLS

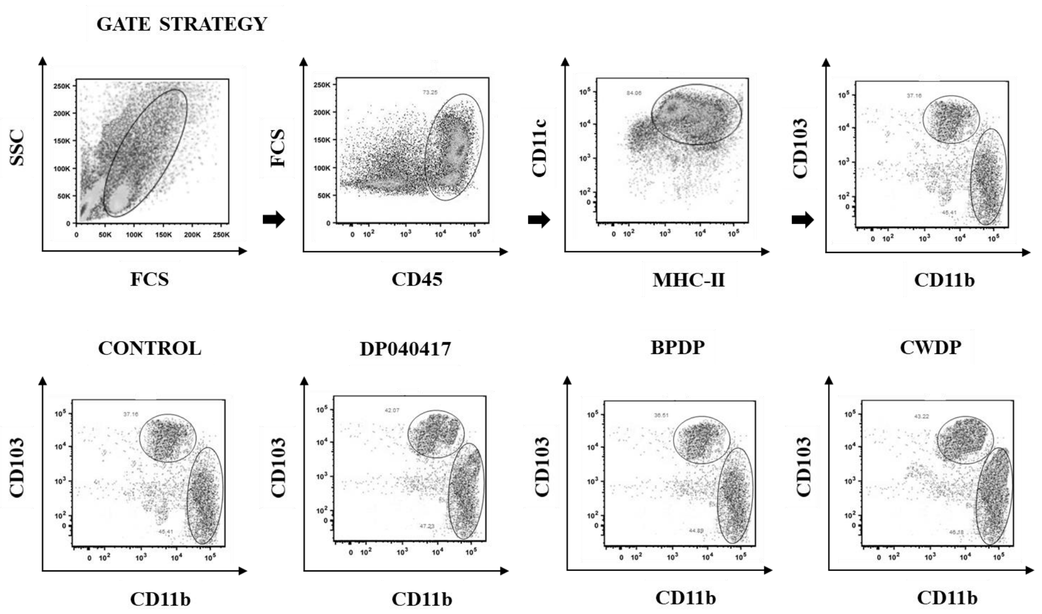

Supplementary Figure 4. Effect of *Dolosigranulum pigrum* 040417 on respiratory dendritic cell populations. Viable (DP), bacterium-like particles (BPDP) or cell wall (CWDP) from *D. pigrum* 040417 were nasally administered to infant mice during five consecutive days. Non-treated infant mice were used as controls. The numbers of MHC-II$^+$CD11c$^+$CD11b^{low}CD103$^+$ and MHC-II$^+$CD11c$^+$CD11b^{high}CD103$^-$ dendritic cells in lungs were determined by flow cytometry one day after the last treatment. Experiments were performed with 5–6 mice per group. The results represent data from three independent experiments.

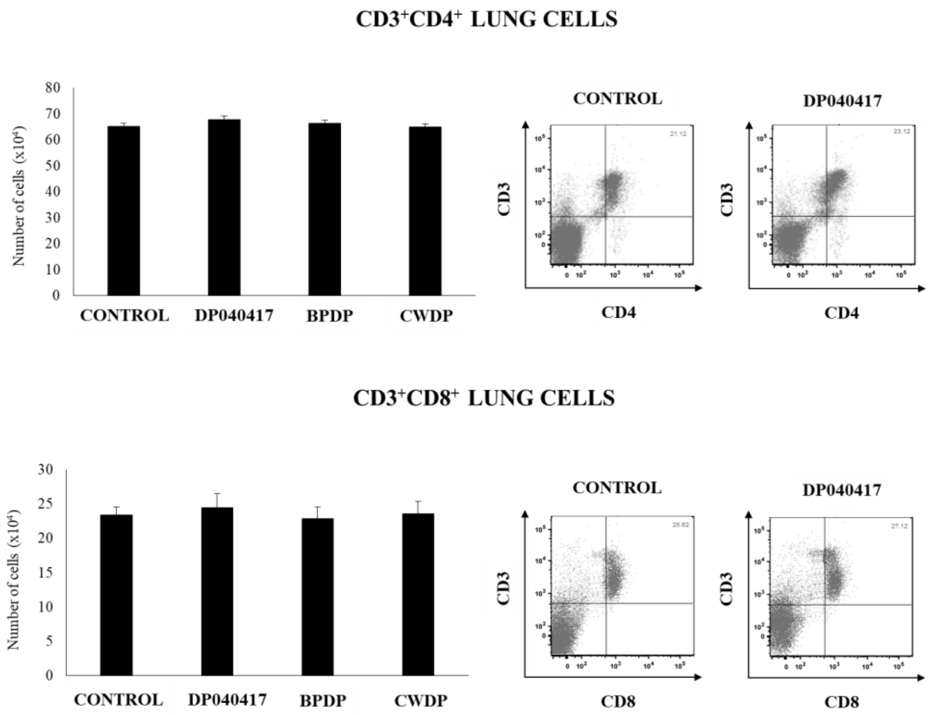

Supplementary Figure 5. Effect of *Dolosigranulum pigrum* 040417 on respiratory T cell populations. Viable (DP), bacterium-like particles (BPDP) or cell wall (CWDP) from *D. pigrum* 040417 were nasally administered to infant mice during five consecutive days. Non-treated infant mice were used as controls. The numbers of $CD3^+CD4^+$ and $CD3^+CD8^+$ T lymphocytes in lungs were determined by flow cytometry one day after the last treatment. Experiments were performed with 5–6 mice per group. The results represent data from three independent experiments.
